# Snake Bite and Dengue: A Twin Tragedy

**DOI:** 10.7759/cureus.19097

**Published:** 2021-10-28

**Authors:** Satish Mahajan, Akhilesh Annadatha, Dhruv Talwar, Vankadari Venkata Sesha Satya Sagar, Anuj R Varma

**Affiliations:** 1 Department of Medicine, Jawaharlal Nehru Medical College, Datta Meghe Institute of Medical Science (Deemed to be University), Wardha, IND

**Keywords:** neuroparalytic snake bite, viral infection, tropical disease, snake bite, dengue fever

## Abstract

Dengue is a viral infection caused by the arboviridae family of viruses and is transmitted by the vector, a mosquito with the scientific name Aedes egyptii. The fever caused by the Dengue virus is best labelled as Break Bone fever because of the severe myalgia that accompanies the infection. Snakebite is also a global health problem. Mostly seen in tropical countries and countries with agriculture as the backbone of the economy, it has a varied presentation in such extremes that it can go from a very mild course of disease not requiring antidote administration to life-threatening complications of respiratory muscles paralysis, coagulopathy, and rhabdomyolysis leading to acute kidney injury based on the nature of the venom of the snake. Here, we report a case of snakebite who was also battling a concurrent Dengue infection thus complicating the management of the patient.

## Introduction

Dengue is a mosquito-borne viral infection usually seen in the tropical regions mostly with also a small percentage in the subtropical areas. Mostly seen in the monsoons, the disease has a typical presentation of fever with rash, body ache, joint pain, headache and can complicate to a stage of hemorrhage or shock and could ultimately lead to death [1]. With such varied presentation, the treatment of the viral infection is tricky. Thrombocytopenia and low white cell count is a common phenomenon encountered. Thrombocytopenia can be mild in some cases which generally doesn’t require transfusion of platelets and can be severe in some cases to the extent that platelets have to be transfused to the patients and failure to do so can result in serious haemorrhagic manifestations resulting in mortality. There have been many neurological manifestations seen with dengue fever, namely, Guillain Barre Syndrome, myositis, encephalitis, and neuropathies [2,3]. Snakebite has become a major public health issue in the tropics and sub-tropics [4]. With many species of snakes present in nature, not all snake bites have harmed humans. The poisonous snakes, however, have caused mortality to their victims by either damaging the muscles or damaging the nervous system or by acting on the blood by deranging the normal clotting mechanisms causing mortality. India has the highest deaths caused due to snake bites in the world, more so in the rural areas [5]. Delay to reach medical services and ignorance of primary care to treat snake bites have been the main contributing factors to the increase in deaths in India [6]. The common venomous snake species encountered in India include Cobra, Krait, Saw scaled Viper, and Russel’s Viper. The clinical signs and symptoms that are usually seen post snake bite are Local edema, bleeding diathesis, pain abdomen, vomiting. Acute neuromuscular weakness with respiratory involvement is the most important neurotoxic effect in a neuroparalytic snake. Symptom evolution and recovery, patterns of weakness, respiratory involvement, and response to antivenom and acetylcholinesterase inhibitors are variable and seem to depend on the snake species, type of neurotoxicity, and geographical variations [7]. The challenge in treating a case of neuroparalytic snake bite with co-existing Dengue infection is the presence of bleeding manifestations secondary to dengue that can act as a barrier for effective treatment of the snake bite. The presence of a neuroparalytic snake bite itself presents as an indication for ventilator support in presence of respiratory muscle involvement and the presence of infections causing thrombocytopenia like Dengue can make the intubation difficult and may lead to bleeding during the same. 

## Case presentation

**Figure 1:**
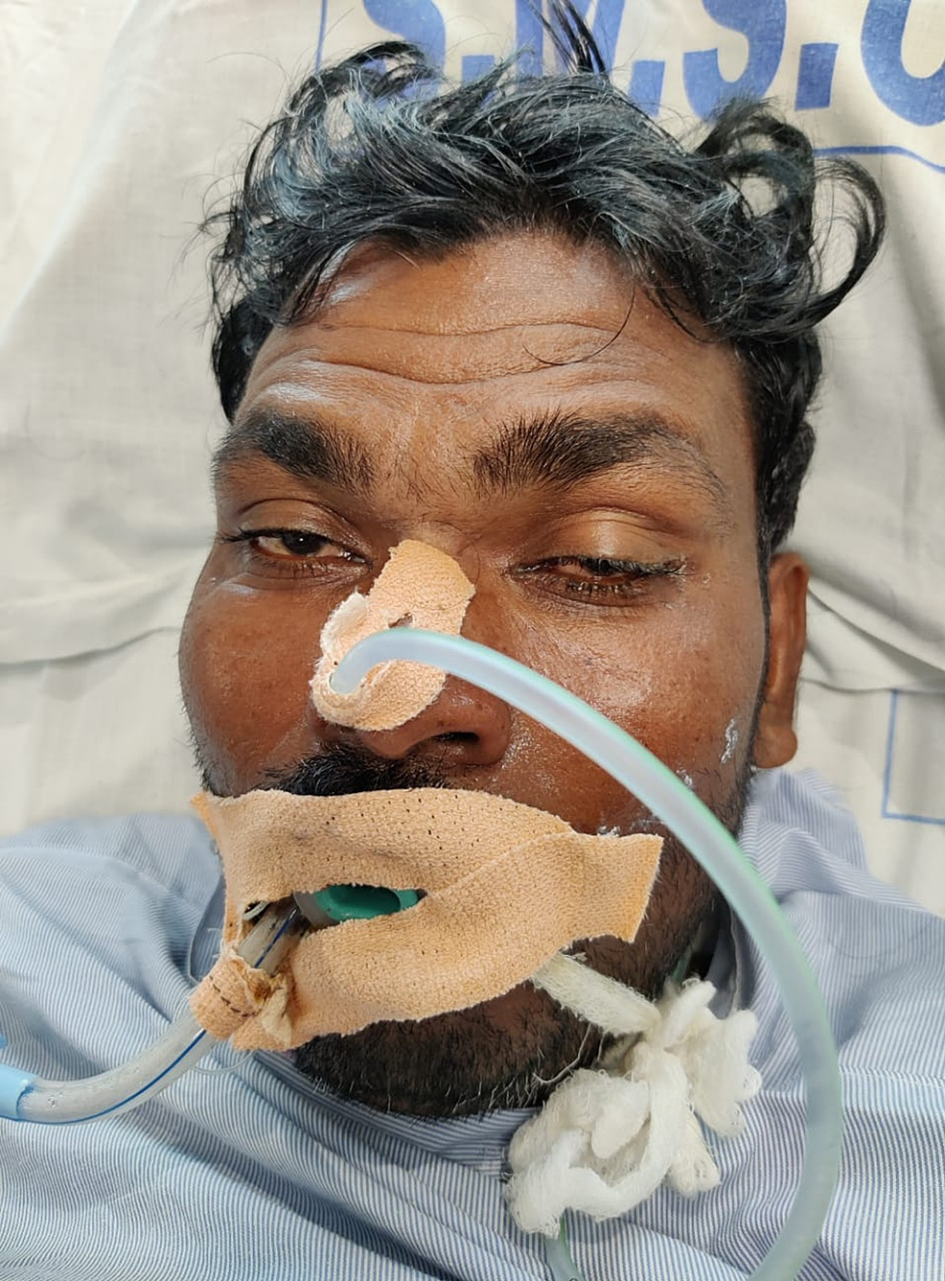
Ptosis in the patient.

A 24-year-old male presented to our hospital with a history of snakebite on the left foot between the great toe and second toe. Post snake bite, the patient had two episodes of vomiting. The patient also had a history of fever for the past three days, which was intermittent, high grade, and was associated with severe body ache. On presentation, the patient had ptosis (Figure 1). The single breath count of the patient was 22. On admission, the patient was febrile with a temperature of 103 Fahrenheit, pulse was 82 beats per minute, respiratory rate was 24 cycles per minute and blood pressure was 100/70 mmHg in right arm supine position, Oxygen saturation was 95% on room air. Within 45 minutes of admission, the single breath count of the patient reduced to 12 and the respiratory rate increased to 32 cycles per minute, Oxygen saturation dropped to 88% on room air. In view of ptosis, reduced single breath count, tachypnea and decreased Oxygen saturation, the patient was intubated and was put on mechanical ventilator. As the snake species could not be identified by the patient and with the signs and symptoms pointing towards a neurotoxic snake bite., the patient was started on treatment for the same. On admission, the lab investigations of the patient revealed a platelet count of 15,000 cells/cu.mm, hemoglobin was 17.2 gm%, white cell count was 8100 cells/cu.mm, hematocrit was 48.6. coagulation profile, lactate dehydrogenase, kidney function tests and total creatinine phosphokinase were unremarkable. The serologies for Dengue, Scrub typhus, and Leptospira were sent and the patient was tested positive for Dengue NS1 Antigen. The patient did not present with any petechiae and the tourniquet test was also found to be unremarkable. The patient was started on intravenous fluids, Paracetamol, and was transfused with Single Donor Platelets (SDP) during his hospital stay by regular monitoring of platelets. Hemoglobin and platelet values of the patient throughout the stay are mentioned in Table 1. The patient was started on Anti Snake Venom (ASV). He was given 20 units of ASV initially on presentation and the patient was monitored for the signs of neurological improvement. The patient was given 20 units of ASV every six hours till the next 48 hours when neurological symptoms improved. The patient had improvement of ptosis, respiratory distress and was gradually weaned off from the Ventilatory support and after five days of admission, he was extubated and was put on oxygen support. The patient was weaned off from oxygen support in the next four days and was monitored for any delayed neuroparalytic effects of the snake bite. The patient was discharged after he was symptom-free for the next seven days.

**Table 1:** Hemoglobin and platelet values of the patient. Platelet count normal range: 150000/cu.mm to 400000/cu.mm. Hemoglobin normal range: 13.8 to 17.2 gm/dl.

Day	Hemoglobin	Platelet count	Transfusion
Day 0	17.2 g/dl	15000/cu.mm	1 unit of SDP
Day 1	14.1 g/dl	25000/cu.mm	-
Day 2	11.8 g/dl	18000/cu.mm	1 unit of SDP
Day 3	8.5 g/dl	117000/cu.mm	1 unit of packed red cells
Day 8	10.7 g/dl	265000/cu.mm	-
Day 14	11 g/dl	405000/cu.mm	-

## Discussion

**Figure 2:**
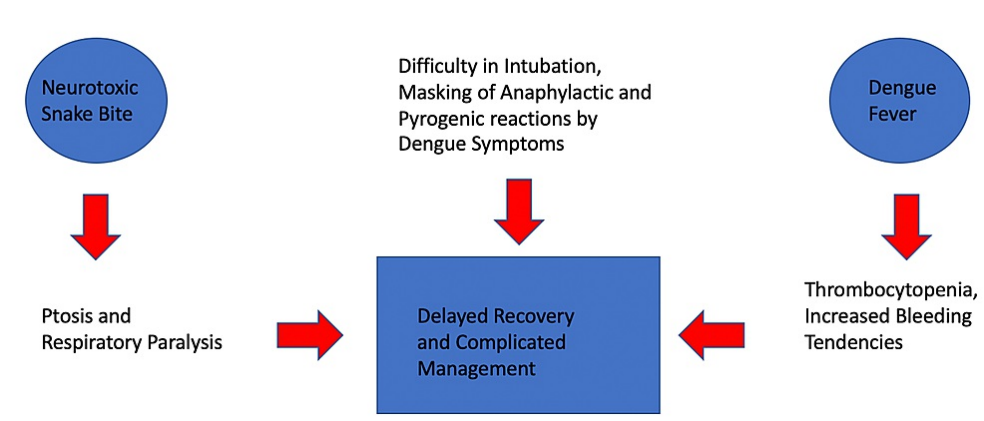
Interaction of dengue fever and snakebite in our case.

Neuroparalytic snake bites have been known to cause a great deal of morbidity and mortality if not treated promptly. As a majority of primary health care centers have no access to ventilator support, the patients requiring ventilatory assistance will have to be referred to centers with access to the same. In absence of ventilators, there is a limited role of administering neostigmine and atropine. As the physiology of a neuroparalytic snake bite is similar to myasthenia, theoretically acetylcholinesterase inhibitors are effective in the treatment of such cases. Atropine is added to neostigmine to negate the nicotinic effects of neostigmine. Neostigmine is used as an adjunct to the Anti Snake Venom. Some studies have shown that edrophonium test can be used to predict the response of treatment with neostigmine [8]. There are several reports of central effects such as drowsiness, coma, and loss of brainstem reflexes following snakebite. Many of them are isolated case reports with poor snake identification [9]. One more challenging aspect of our case was the presence of a Neuroparalytic snake bite with Dengue which made the treatment more complicated. The co-existence of both of these conditions can be explained by the presence of our hospital in the tropical region of central India along with the monsoon season as both the illnesses are favored by stagnant water. Dengue is characterized by thrombocytopenia which can cause bleeding manifestations which can prove to be lethal specially in the case we report as the patient was already neurologically compromised. Dengue can also mask the pyrogenic and anaphylactic reactions of the ASV that is needed to be administered in snake bite cases as the symptoms of these reactions are similar to the symptoms of dengue fever which includes high-grade fever with chills, rashes and myalgia (Figure 2). Administration of platelets below critical levels has been the cornerstone of treatment along with adequate hydration which was followed in our case. Our patient's recovery was delayed as a result of the synergistic effect of dengue fever with a snake bite. While treating the neurological manifestations of snakebite the severe thrombocytopenia caused by dengue fever also had to be dealt with caution. Our patient received multiple platelet transfusions as well anti-snake venom to cover both the aspect of his condition which were managed successfully. Thus, the treating physicians specially those catering to the patients in the tropical regions, have to be vigilant in treating such cases with contrasting presentations of hemolytic and neurotoxic pictures. Both the diseases require equal attention of the clinician and have to be treated simultaneously rather than preferring one over the other such as in our case.

## Conclusions

Neuroparalytic snake bite is in itself a dreadful condition. Dengue fever is also a disease causing a great deal of morbidity and mortality. The presence of both the conditions have been reported rarely making this an important case to report.
